# The interplay between experiential and traditional learning for competency development

**DOI:** 10.3389/fpsyg.2015.01305

**Published:** 2015-09-02

**Authors:** Sara Bonesso, Fabrizio Gerli, Claudio Pizzi

**Affiliations:** ^1^Department of Management, Ca' Foscari University of VeniceVenice, Italy; ^2^Department of Economics, Ca' Foscari University of VeniceVenice, Italy

**Keywords:** competency development, emotional social and cognitive competencies, learning methods, traditional learning, individual experiential learning, social experiential learning

## Abstract

Extensive research demonstrated that firms may pursue several advantages in hiring individuals with the set of emotional, social, and cognitive (ESC) competencies that are most critical for business success. Therefore, the role of education for competency development is becoming paramount. Prior studies have questioned the traditional methods, grounded in the lecture format, as a way to effectively develop ESC competencies. Alternatively, they propose experiential learning techniques that involve participants in dedicated courses or activities. Despite the insights provided by these studies, they do not take into account a comprehensive set of learning methods and their combined effect on the individual's competency portfolio within educational programs that aim to transfer primarily professional skills. Our study aims to fill these gaps by investigating the impact of the interplay between different learning methods on ESC competencies through a sample of students enrolled in the first year of a master's degree program. After providing a classification of three learning methods [traditional learning (TL), individual experiential learning (IEL), and social experiential learning (SEL)], the study delves into their combined influence on ESC competencies, adopting the Artificial Neural Network. Contrary to prior studies, our results provide counterintuitive evidence, suggesting that TL needs to be implemented together, on the one hand, with IEL to achieve a significant effect on emotional competencies and, on the other hand, with SEL to have an impact on social competencies. Moreover, IEL plays a prominent role in stimulating cognitive competencies. Our research contributes to educational literature by providing new insights on the effective combination of learning methods that can be adopted into programs that transfer technical knowledge and skills to promote behavioral competencies.

## Introduction

Research has broadly acknowledged that behavioral competencies, namely emotional, social and cognitive (ESCs) competencies, account for a substantial and important amount of the variance in predicting job performance, career success, and personal wellbeing (Boyatzis, 1982; Spencer and Spencer, 1993; Goleman, 1998; Williams, 2008; Amdurer et al., 2014). Consequently, firms may pursue several advantages in hiring individuals with the set of ESC competencies that are most critical for their business success (Robles, 2012), such as having shorter induction period, higher productivity, innovative performance, and organizational commitment. Although employers call for highly skilled candidates with a competency profile characterized not only by professional skills but also by behavioral competencies, prior research shows that newly hired employees do not possess ESC competencies at the level that employers desire (Azevedo et al., 2012). Research has also highlighted that this competency shortage inhibits solid growth and the development of new ventures (Boyles, 2012).

Behavioral competencies assume an even more crucial role for high managerial positions. In this regard, research suggests that the effectiveness of leaders tend to be related to their level of possession of ESC competencies (Goleman et al., 2002; Young and Dulewicz, 2007; Hopkins and Bilimoria, 2008; Koman and Wolff, 2008; Walter et al., 2011). Therefore, to better prepare individuals for their roles as responsible leaders in creating and managing sustainable businesses, scholars advocate that ESC competencies need to be developed in concert with professional skills (Rasmussen et al., 2011). Prior studies show that by designing specific courses or focusing on a set of competencies in selected courses (Chen et al., 2004; Boyatzis and Saatcioglu, 2008; McEnrue et al., 2009; Sheehan et al., 2009; Waddock and Lozano, 2013), and assigning specific experiential activities assigned during the course (Vaatstra and De Vries, 2007; Hoover et al., 2010; Landau and Meirovich, 2011; Kuijpers and Meijers, 2012) stimulates the development of emotional, social, and cognitive (ESC) competencies. These contributions have questioned the traditional lecture format as a way to effectively develop ESC competencies. Alternatively, they propose experiential learning techniques that involve participants into reflection, interactive engagement and practical experience simultaneously activating “cognitive, behavioral, and emotional dimensions of learning and behavioral change necessary for skill acquisition” (Hoover et al., 2010: 195). The involvement in such dedicated courses and experiential exercises requires personal responsibility since the change is self-directed, in line with the pedagogy of whole person learning.

Despite the well-founded importance of the relationship between the use of specific teaching methods and competency development, studies that delve into it still present some limitations. First, they do not take into account a comprehensive set of both traditional and experiential learning methods that may stimulate the individuals to perform behaviors with the intent to activate specific ESC competencies. Indeed, research has mainly focused on experiential learning approaches, whereas the effect of traditional in-class training on ESC competencies remains controversial. Second, extant literature has primarily focused the attention on the influence of a specific course or learning activity, neglecting the investigation of a combined or complementary effect of traditional and experiential learning methods on the individual's competency portfolio. Finally, as highlighted by prior research (Cherniss et al., 2010), the aforementioned competency development programs require a high level of investment for the organizations since they need to be fairly intensive in terms of time and effort devoted by the participants. Including ESC competency development as learning objectives in typical courses may help to reduce the difficulties associated with the delivery of these types of educational programs and may offer the opportunity for participants to learn technical knowledge and skills jointly with behavioral competencies. So far, empirical research has not investigated if the use of specific learning methods within educational programs that aim to transfer primarily professional skills may also stimulate ESC competencies. Our study aims to fill these gaps by addressing the following research question: Which combination of different learning methods may contribute to develop ESC competencies within educational programs that aim to endow participants primarily with technical knowledge and skills?

Our exploratory study, carried out in a public university located in northern Italy, involved students enrolled in the first year of master's degree courses in different disciplines (economics, management, humanities, languages, cultural heritage and restoration, scientific and technological areas). After providing a classification of different learning methods [traditional learning (TL), individual experiential learning (IEL), and social experiential learning (SEL)], we investigated their combined influence on ESC competencies development.

This paper is organized as follows. In the following section, we provide a literature review on competency development, formulating the research hypotheses on the combined effect of different learning modes on ESC competencies. Next, in the methods section, we illustrate the setting and the method adopted in this study, namely artificial neural networks. Afterwards, we present the results of the empirical analysis, and, in the final section, we discuss the implications and avenues of future research.

## Theoretical background and research hypotheses

A behavioral competency is defined as “the underlying characteristics of a person that lead to or cause effective and outstanding performance” (Boyatzis, 1982: 21). Prior studies identified three main clusters of competencies that distinguish outstanding performers from average ones across countries and jobs (Goleman, 1998; Boyatzis, 2009; Emmerling and Boyatzis, 2012): emotional competencies (the ability to recognize, understand, and use emotional information about oneself), social competencies (the ability to recognize, understand and use emotional information about others), and cognitive competencies (the ability to think or analyze information and situations).

Criticism has been recently raised against management education, claiming it has been unable to provide the necessary behavioral competencies for leaders and managers to accomplish their complex agendas. Specifically, it has underlined the need for education to renew its orientation toward self-awareness and interpersonal skills and not just learning concepts and techniques (Waddock and Lozano, 2013). However, teaching behavioral competencies is more difficult than teaching technical knowledge because instructors must engage and interact with students, more than in typical lecture approaches. The extant literature has provided some insights into these types of learning environment and teaching methods that stimulate the development of behavioral competencies.

The study of Boyatzis and Saatcioglu (2008) showed that the participation in a specific course designed according to the intentional change theory (Boyatzis, 2006)—which implies activities about defining dreams and aspirations for the future, assessing current individual competencies, detecting personal strengths and weaknesses, writing a learning plan, and practicing new behaviors—significantly improved participants' ESC competencies. Other studies focused on a specific set of competencies in selected courses. For instance, Chen et al. (2004) showed that social skills like teamwork may be improved by designing a course that not only favors conceptualization or transfer of declarative knowledge about teamwork but also elaborates this knowledge and transforms it into proceduralized skills and abilities through in-class and out-of-class group exercises. In their research, McEnrue et al. (2009) investigated the impact of an educational program designed to develop emotional competencies. The program encompassed activities, such as (a) writing a self-development plan, (b) being involved in coaching sessions and meetings with the instructor as a source of feedback and assistance, (c) performing role-playing exercises that permit practice, observation, and learning about the competencies, (d) interviewing professionals who master emotional skills and provide feedback to other participants who criticize their development plan, (e) reading and viewing film clips, (f) keeping a weekly journal, and (g) writing a summary statement in which participants identified the learning they had achieved. In another study, through the design of a course that simulated a functioning organization, Sheehan et al. (2009) provided evidence of the positive impact of classroom-as-organization approach and complementary experiential methods (journal writing, one-on-one meetings, and the exit interview) to stimulate acquisition of emotional and social competencies.

Other studies, instead of investigating the impact of specific programs on competency development, considered the impact of some experiential learning environments or activities assigned during the course. For instance, within a required coursework, Hoover et al. (2010) analyzed the effect of several experiential exercises on the development of specific competencies (communication, teamwork, leadership and initiative, decision making, and planning and organizing). In their study, Vaatstra and De Vries (2007) found that the more individuals are involved in problem-based or project-oriented learning environments the more often they acquired behavioral competencies than students who completed the traditional type of education. Similarly, Kuijpers and Meijers (2012) demonstrated that practice- and inquiry-based educational programs encourage participants to deploy and foster behavioral competencies. The relevance of the learning environment in promoting competency development has been further underlined by Landau and Meirovich, who showed that “in classes where professors encourage participation through teamwork, discussions, debates, simulations, and role playing, students are more likely to need to rely on their emotional competencies, than in traditional classroom settings where the “instructor is on stage” (2011: 92). According to the authors, a participatory class environment allows interaction with the instructor and peers providing opportunities to read and understand emotions. All these studies suggest a need for stronger emphasis on the use of experiential learning techniques in training programs that aim to promote the development of ESC competencies, since the traditional methods—with their focus on a mere knowledge acquisition—are not enough. Indeed, the action-based nature of behavioral competencies suggests that their development may require didactic approaches that move beyond traditional lectures, discussions, and exam formats (Bay and McKeage, 2006). According to this literature, the TL environment does not represent an opportunity for competency improvement since it provides declarative knowledge and a set of general conceptual and analytical skills, but it does not enable students to learn and practice a set of behavioral repertoires. To date, research has proposed a distinction between soft, behavioral skills training and hard, technical skills training, in terms of pedagogical tools, associating the former with experiential learning approaches and the latter with traditional methods (Laker and Powell, 2011).

Despite these insights on the relationship between specific teaching methods and the development of competencies, a limit of these studies is that they do not take into consideration the possible combined effect of traditional and experiential learning methods on behavioral competencies (Vila et al., 2012). Moreover, they analyze courses or learning activities designed *ad hoc* for behavioral competency development, and they do not consider that ESC competencies may also be stimulated in educational programs primarily devoted to endow participants with technical skills (Boyatzis et al., 2002). Dedicated courses require an intense effort in terms of time and motivation by the participants as well as conspicuous economic investments by their organizations. In addition, barriers related to the implementation of dedicated emotional and social competencies training have been previously discussed in the literature (Lindebaum, 2009; Waddock and Lozano, 2013; Bedwell et al., 2014). Furthermore, many academic programs do not have the flexibility to integrate dedicated ESC competencies courses into already heavy curricular loads. Therefore, including ESC competencies as learning outcomes in existing programs that are primarily devoted to transfer technical skills and involving participants in pedagogical methods that stimulate their acquisition may provide the opportunity to acquire and practice behavioral competencies, while reinforcing the educational aims of the programs.

Besides revising the objectives and the learning methods of existing courses, complementing technical training with competency development calls for a change from the pedagogical point of view. Indeed, instructors require different teaching skills and tools as well as the acquisition of new attitudes and mindsets in order to integrate their traditional role of “expert” with a the new role of “learning facilitator.”

Drawing on management education literature (Wertenbroch and Nabeth, 2000; Hawtrey, 2007; Michel et al., 2009; Vila et al., 2012; Frost and Wallingford, 2013; O'Leary and Stewart, 2013; Bedwell et al., 2014), we identified several types of learning methods that can be categorized as follows:

- Traditional Learning methods (TL): These refer to those teaching methodologies (such as in class lessons and talks from visiting experts during class) that present a high-level theory of the field to the students. Individuals are then expected to solve problems by applying the theory to a specific context.
- Individual Experiential Learning methods (IEL): These refer to methodologies (such as simulations and individual projects) in which individuals are able to experiment and progressively discover knowledge and apply skills in a domain by trial and error.
- Social Experiential Learning methods (SEL): These refer to methodologies (such as group assignment, business game) in which individuals acquire knowledge and skills by interacting with others and sharing their experiences.

The above-mentioned categories have different conceptions of the learning process and the related pedagogical methodologies. As highlighted by Higgins and Elliott (2011: 346–347) “the traditional approaches to learning make the assumption that knowledge must be transmitted and received in the form of explicit information, after which learners can apply this new found knowledge to their own purposes. In this case learning is viewed as an external objective process.” Dhliwayo (2008) argues that traditional passive learning methods can only help the students to memorize the concepts and theories that are taught to them. Thus, those who are exposed to such learning strategies are simply spectators rather than active participants. Experiential learning via individual and social practice and reflection requires a more pro-active approach. Experiential learning is described as a progression of sequential stages (direct experience, reflective observation, abstract conceptualization, and active experimentation) in a cycle that generates personal interpretations of new events (Kolb, 1984). Similarly, Jennings and Wargnier (2010) suggest four elements of experiential learning: (1) the exposure to experiences, (2) the practice and the embedding of experiences, (3) conversation, and interaction with others, and (4) regarding the experiences to make further sense and reflections on what we do, see, and hear. Engaging individuals in experiential learning means to create the opportunity to stimulate them toward individual action. Indeed, experiential learning is conceived as “a holistic process of adapting to the world that requires the integrated functioning of the total person, which includes thinking, feeling, perceiving, and behaving, as well as interactions between the person and the environment” (Ng et al., 2009: 513). As demonstrated by Morris et al. (2013: 449) in a study that analyses the impact of specific problem-solving tasks and learning outcomes, “when placed in a context that centers on experiencing and performing real tasks that support entrepreneurial outcomes, students appear to get better at demonstrating specific competencies.”

Experiential learning methods ascribe a prominent role to the reflection mechanism that is relevant for the recognition and understanding of emotional information about oneself, namely emotional competencies. Specifically, IEL techniques allow individuals to apply theories through concrete actions, gain feedback to assess the possible deviations from desired objectives, confirm or disconfirm their assumptions and understandings, and, consequently, mold their behaviors into competencies. Moreover, IEL activities may encourage individual reflection on their own emotions through elaboration of individual project works, structured presentations to class or seminars and workshops, labs, and field works, along with self-assessment techniques, such as e-learning and testing laboratories. Indeed, these activities facilitate opportunities for students to think on and derive meaning from their experiences, which represent a necessary element in developing emotional competencies. Individuals involved in IEL may become more conscious about their strengths and weaknesses and may be prone to define standards of excellence, to act on opportunities, and to handle change—all behaviors ascribed to the emotional competencies. For instance, the engagement in individual project works or laboratory activities requires individual to set measurable goals and respect deadlines as well as adapt ideas and actions based on new information emerging from the practical experience; in such a way they nurture achievement orientation, self-control, and flexibility. On the other hand, SEL may have a limited impact on emotional skills development. When individuals are involved in activities or exercises that require interpersonal exchange or group work activities, they may be more concentrated in handling the relationship in order to achieve the task assigned within the deadline, instead of learning how to reflect and manage their own emotions. Concerning TL techniques, prior research has argued that lectures provide the content basis of declarative and procedural knowledge concerning technical skills, but their effect on behavioral competency development has been questioned. We argue that the adoption of some TL may also allow participants to become aware of the relevance of emotional competencies, even though the use of these techniques is not explicitly related to the acquisition of these competencies in existing courses. For instance, the use and discussion of films and video clips, participation in seminars, or attending expert talks as auditors may represent an opportunity not only to observe examples of application of technical tools but also to see the demonstration of effective (or ineffective) displays of emotional skills by others, such as striving to improve or meeting a standard of excellence, acting on opportunities congruently with one's values, or adapting plans, behaviors, or approaches to fit major changes. Therefore, we expect that:

*Hypothesis 1*. The more individuals are involved in IEL and TL the more they possess emotional competencies.

Experiential learning that offers opportunities for social interactions, such as in-class group discussions, group project works, role playings, and participation in online forums allow students to practice “real-life” situations that require them to become aware of the emotions of others and handle them. As maintained by Landau and Meirovich (2011: 92) in their study on the impact of a participative class environment on emotional intelligence, “Interaction with the instructor and peers gives students the opportunity to read emotions, see how emotions might create intragroup problems, incorporate their emotions into thought, and control their emotions and the emotions of others.” Indeed, these techniques may positively influence social competencies since they put individuals in situations that require solving conflicts; they must convince others, relate well to people of diverse backgrounds, and work well in teams by being respectful of others. On the other hand, IEL may have a limited impact on social skills development. The only opportunity for students to recognize, understand, and use social information about others is by observing the actions of peers and related outcomes, for instance, during class presentations or when they interact with lecturers during the execution of assigned tasks. Prior studies maintained that in traditional classroom settings (TL) the “instructor is on stage” (Landau and Meirovich, 2011: 92), and the opportunity for participants to understand and manage others' emotions is limited. Nevertheless, the positive effect of SEL may be reinforced by TL techniques like using and discussing multimedia materials or attending seminars or conferences. Indeed, the observation and discussion of non-verbal and verbal communication through lectures or expert talks may help student to sense others' feelings and perspectives, to understand people with different backgrounds and culture, to see how individuals use tactics for persuasion, or to send clear and convincing messages to an audience. Hence, we expect that:

*Hypothesis 2*. The more individuals are involved in SEL and TL the more they possess social competencies.

The competency-based research has shown that two cognitive competencies (systems thinking and pattern recognition) repeatedly demonstrate to differentiate effective job performance across different professional roles (Boyatzis, 2008, 2009). The development of such cognitive competencies occurs when the learning process changes the individuals' knowledge domain, for example, when gaining new knowledge or developing a more sophisticated mental model of a specific subject. *Pattern recognition* is the capability of recognizing patterns or trends in random information, events, or situations. It is an extremely important capability that allows individuals to learn from the past and to identify commonalities or similarities among various and often very different situations. On the other hand, *systems thinking* is the ability to identify the many and various factors that affect a complex situation or event and recognize both the causes and effects of actions and outcomes. This competency allows individuals to explain interactions among factors using, for instance, diagrams, flow charts, or detailed but simple discourses (Baron, 2006; Boyatzis, 2009).

Through individual (IEL) and social (SEL) learning methods individuals have the opportunity to acquire new knowledge and distill their observations and reflections into more general concepts and principles. Thus, they are more likely to add elements to their knowledge structures and build new connections between knowledge elements, facilitating the recognition of patterns. Furthermore, individual and social assignments, such as project work presentations and discussions or laboratory activities, may spur individuals to approach information processing and analysis as well as the explanation of phenomena through the identification of the various factors that affect situations or events. We also maintain that TL may stimulate cognitive competencies through the absorption of information from an expert (the instructor or invited lecturer). Listening and interacting with an expert may not only provide knowledge acquisition about a specific topic but may also favor new associations and similarities among concepts learned in prior learning experiences and the understanding of cause and effect relationships that explain events and situations. Therefore, we expect that:

*Hypothesis 3*. The more individuals are involved in IEL, SEL, and TL the more they possess cognitive competencies

## Method

### Sample and procedure

We carried out the empirical research in a public university located in northern Italy that has approximately 20,000 students enrolled in four broad scientific and cultural areas: economics, foreign languages and literatures, humanities and sciences. The sample of students involved all those enrolled in the first year of five different master's degree courses in the academic year 2011–2012 (business administration, economics and management of arts and cultural activities, computer science, international relations, global development, and entrepreneurship). These programs were chosen because of their variety and because of the presence of non-conventional teaching methodologies (that is, not only frontal lectures).

We set up a self-evaluation questionnaire to be filled in by each of the analyzed students at the end of the first semester of the first year of their master's degree program. The questionnaire, administrated online, was made up of two sections. In the first one we collected data on the level of possession of students' ESC competencies, asking students to rate themselves on a five-point scale, according to the Emotional Competence Inventory–University version (ECI-U). In the second section of the questionnaire, we gathered information on the learning methods students had been involved with during the academic courses they had attended in the first semester of the academic year and on a set of variables that could have influenced the student's competency portfolio. The questionnaire was tuned by applying a pilot test to a small sample of students not involved in the large-scale analysis. The population of students enrolled in these five courses was made up of 240 students, 45% of whom completed our questionnaire. All students gave explicit consent to participate in the research. All the authors are subject to the Ethical Code of their affiliating Institution (Ca' Foscari University of Venice), and all protocols governing the use of human subjects were followed. The research protocol was approved by the Scientific Committee of the Ca' Foscari Competency Centre, under which this research has been conducted.

To avoid the biases due to the selection process of respondents, we tested if the sample of respondents was statistically representative of the whole population of students enrolled in the five courses under analysis by considering some personal variables such as gender, age, type of course, the University in which they pursued their bachelor's studies, and evaluations obtained at the bachelor's degree and at high school level. Concerning the gender, the sample is composed by 24.07% of males and the remaining 75.93% of females, whereas the non-respondents are 34.85% males and 65.15% females. The respondents were 24.15 years old on average (*SD* = 1.08) and the non-respondents were 25.33 years old on average (*SD* = 3.85). As regards the different master's programs in which the students were enrolled, the majority of the respondents belonged to the business administration (39.81%) and to the management of arts and cultural activities courses (31.48%), followed by global development and entrepreneurship (12.96%), international relations (1.11%), and computer science (4.63%). Considering the non-respondents, 37.12% were enrolled in management of arts and cultural activities, 24.24% in business administration, 20.45% in international relations, 9.85% in global development and entrepreneurship, and 8.33% in computer science. We also considered in which university the students pursued their bachelor's degree, and the 52.78% of the respondents attended it in the same University they were enrolled for the master's course (49.24% for non-respondents) whereas 47.22% came from a different University (50.76% for non-respondents). As far as the performance of the students, we took into account the evaluations obtained at the bachelor degree and high school levels. As regard the grade obtained at the end of the bachelor's degree program, the two groups of students showed very similar grades (respondents: mean = 101.21 out of 110, *SD* = 6.51; non-respondents: mean = 101.07, *SD* = 7.45). Finally, the respondents achieved on average 83.56 out of 100 (*SD* = 11.62) as high school final grade, whereas non-respondents showed a slightly lower evaluation (mean = 80.74; *SD* = 11.73).

We used the chi-square test of independence to determine whether the type of respondents (students included in the sample and non-respondents) is related to gender (3.284, *p*-value = 0.07); type of course (10.081; df = 4; *p*-value = 0.039) and previous University (0.297; df = 1; *p*-value = 0.586); and we used the *t*-test and the Kolmogorov-Smirnov test for age (*t* = 3.100, *p*-value = 0.002; *z* = 1.505, *p*-value = 0.022), the evaluations obtained at bachelor's degree level (*t* = 0.148; *p*-value = 0.882; *z* = 0.670; *p*-value = 0.761) and the evaluations obtained in high school (*t* = −1.818, *p*-value = 0.70; *z* = 1.165, *p*-value = 0.133). These results show that there are no significant differences between the sample of respondents and the non-respondents, except for the age and the type of course they followed, which was slightly biased toward the students enrolled in the course in business administration. We restricted the original sample of 108 respondents to a sample of 95 students since we excluded part-time students and full-time students who did not attend, either partially or totally, the academic courses during the period under investigation from the subsequent analysis. This is because in our analysis we considered the relationship between ESC competencies and the didactic methodologies adopted in the course, and, consequently, the inclusion of students who did not experience the learning approaches under investigation could have biased our analysis.

### Dependent variables

The ESC competencies were measured using the ECI-U questionnaire, which encompasses 24 competencies that are described through three behavioral indicators. Students were asked to rate themselves, according to the frequency they demonstrated each of the 72 behavioral indicators during the previous 6 months on a scale ranging from zero (never) to 5 (always). The ECI-U is a proprietary instrument and a detailed description is reported in the Appendix in Supplementary Material. This instrument has been developed, implemented, and tested in higher education contexts and adopted in several research settings (Sharma, 2012). The ECI-U showed the desired levels of internal reliability (Wolff, 2005) and convergent validity in confirmatory factor analyses for both theoretical clusters (Goleman et al., 2002; Wolff, 2005) and empirical clusters (Boyatzis and Sala, 2004). In addition, a wide variety of validation studies showed strong and consistent validity in predicting or explaining life and job outcomes (Boyatzis and Sala, 2004; Wolff, 2005).

*Emotional competencies* were measured with the 10 following competencies: Emotional Awareness, Accurate Self-Assessment, Self-Confidence, Optimism, Emotional Self-Control, Trustworthiness, Conscientiousness, Adaptability, Achievement Orientation, and Initiative (α = 0.86). *Social competencies* were measured with the 12 following competencies: Empathy, Organizational Awareness, Service Orientation, Cultural Awareness, Developing Others, Inspirational Leadership, Building Bonds, Influence, Conflict Management, Teamwork, Communication, and Change Catalyst (α = 0.94). *Cognitive competencies* included the final two competencies of Systems Thinking and Pattern Recognition (α = 0.78).

To overcome the limits of the self-evaluation test, we compared the competency assessment of the students with the evaluation provided by their external raters. At the end of the students' questionnaire, we asked each respondent to provide the names and e-mail addresses of three external raters to carry out a multi-rater assessment on students' ESC competencies and to gather a honest and open evaluation based on the ECI-U framework. Prior studies involved external raters to make it possible to compare the self-perception with a third-party evaluation and, consequently, strengthen the assessment process. As an example, some studies used peer evaluation conducted among the course participants or among friends or family members (Van der Zee et al., 2002; Camuffo et al., 2009; Ohland et al., 2012) or bosses and other managers (Furst-Bowe et al., 1995; Scharf and Bell, 2002; Scott and Yates, 2002; Raybould and Wilkins, 2005; Razak et al., 2008) or teachers (Leckey and McGuigan, 1997; Rapisarda, 2002; Jackson and Chapman, 2012) or personal and professional contacts (Boyatzis et al., 2015). Relying on their observations, raters provide an assessment of the frequency of manifestation of students' behaviors, mitigating the issue of social desirability (Paulhus and Reid, 1991) and other possible biases and unreliable responses associated with self-assessment (Dunning et al., 2004). The questionnaire for the external raters consisted of only one section through which we collected the information on the student's competencies, according to the ECI framework. The only additional variable we gathered from the external raters was the kind of relationship they had with the student (friendship, working relationships, etc.). Not all the students provided valid contacts for their external raters and not all of them answered the questionnaires. For the purposes of the comparison between self and external evaluation, we considered only data regarding the students (*n* = 51) who obtained a complete external evaluation. All external raters (*n* = 85) gave explicit consent to participate in the research. Most of them were friends (34%), followed by parents (17.6%), siblings (16.5%), and partners (16.5%); only 11% of the external raters were fellow university students, 3.3% were other relatives, and only 1.1% were co-workers or bosses. We carried out the internal consistency reliability test on the self-ratings of the total sample of students (*n* = 95) as well as on the external ratings (“total others”) aggregated by student and their corresponding students' self-ratings (*n* = 51). The results are presented in Table 1, which compares—through the *t*-test—the self-ratings expressed by the students with the external evaluations (“total others” ratings) expressed about them, showing a high level of overlap between these two perspectives.

**Table 1:** **Comparison between students' self-ratings and external ratings on ESC competencies**.

**ESC competencies**	**Students: Mean score (*n* = 51)**	**Students: Standard deviation (*SD*)**	**External raters: Mean score (*n* = 85)**	**External raters: Standard deviation (*SD*)**	***t*-value**	**Sig. 2-tailed**
Emotional awareness	4.235	0.567	3.915	0.591	2.990^***^	0.004
Accurate self-assessment	4.255	0.493	3.881	0.645	3.671^***^	0.001
Self-confidence	3.444	0.659	3.527	0.646	−0.886	0.380
Emotional self-control	3.758	0.593	3.613	0.573	1.816^*^	0.075
Achievement orientation	3.804	0.508	3.935	0.545	−1.461	0.150
Initiative	3.346	0.589	3.616	0.611	−2.394^**^	0.021
Trustworthiness	4.098	0.594	4.112	0.646	−0.142	0.888
Conscientiousness	4.523	0.500	4.501	0.426	0.272	0.787
Adaptability	3.961	0.564	3.911	0.604	0.327	0.745
Optimism	3.725	0.717	3.734	0.674	−0.078	0.938
Empathy	4.039	0.528	3.938	0.583	1.285	0.205
Service orientation	3.922	0.536	3.920	0.546	0.013	0.989
Organizational awareness	3.902	0.687	4.078	0.641	−1.632	0.109
Cultural awareness	4.222	0.602	4.080	0.669	1.583	0.120
Leadership	3.654	0.683	3.644	0.684	0.165	0.870
Communication	3.686	0.658	3.636	0.648	0.535	0.595
Conflict management	3.386	0.627	3.187	0.790	1.621	0.111
Change catalyst	3.412	0.691	3.347	0.807	0.850	0.399
Influence	3.562	0.685	3.558	0.575	0.037	0.971
Developing others	3.673	0.767	3.736	0.578	−0.589	0.559
Building bonds	3.863	0.775	3.935	0.657	−0.562	0.577
Teamwork	4.013	0.622	4.082	0.608	−0.688	0.494
System thinking	3.353	0.527	3.613	0.586	−2.643^**^	0.011
Pattern recognition	3.242	0.604	3.315	0.756	−0.609	0.545

* p < 0.10;

** p < 0.05;

*** *p < 0.01*.

The only competencies that present significant differences concern the Self-Awareness and the Cognitive clusters. Prior research showed that these competencies are difficult to assess by an external rater either because they are difficult to observe or because they are not easily understandable (Wolff, 2005; Boyatzis et al., 2015). Indeed, these competencies require that the external observers have the opportunity to observe one's own reasoning patterns and feelings accurately. If such an individual does not explicitly describe his or her emotions and cognitive processes, the rater cannot express any accurate assessment. Through this analysis we could conclude that students' self-evaluation on ESC competencies can be considered as reliable and can consequently be used as dependent variables in our model.

### Independent variables

We defined the learning methods starting from the classifications discussed in prior research on educational methods (Wertenbroch and Nabeth, 2000; Hawtrey, 2007; Michel et al., 2009; Vila et al., 2012; Frost and Wallingford, 2013; O'Leary and Stewart, 2013; Bedwell et al., 2014). We also asked the director of each master's degree program to integrate the list with further didactic methods implemented in their courses.

We identified a list of 28 teaching techniques from a questionnaire in which we gathered information on the different techniques involving the students. From the original list, we classified the teaching techniques according to the three conceptual categories discussed in the background literature. We assigned six techniques to the TL category, eight to the IEL, and 15 to SEL.

In our work we considered each of these three sets of items as a battery, forming a Guttman scale. In fact, each item shows a different intensity with its associated construct. During the validation of the scale, if the coefficient of reproducibility was above the suggested threshold of 0.9 (Abdi, 2010), we deleted the items that had a marginal coefficient of reproducibility less than 0.85 (Guttman, 1944, 1950).

The results of this analysis enabled us to identify four TL techniques: lecture, participation in seminars/conferences/festivals/exhibitions as auditors, talks from visiting experts during class, and study tour (coefficient of reproducibility 0.916); four IEL techniques: data collection, presentation and discussion of individual project work, individual laboratorial activities outside class, and e-learning and testing laboratories (coefficient of reproducibility 0.921); and 11 SEL activities: reading, analysis, and discussion of articles and cases in groups in class; elaboration of group project works; discussion of group project works; group exercises during lesson; role playings; group laboratory activities outside class; business games; participation in seminars/conferences/festivals/exhibitions as a speaker; participation in seminars/conferences/festivals/ exhibitions as an organizer; e-learning chat discussion groups; and e-learning forums (coefficient of reproducibility = 0.925). For each student we computed the value of the Guttman scale for the three learning methods in terms of the number of techniques a student was involved in and the maximum number of techniques considered by each learning method.

### Controls

Our study is not longitudinal and for this reason we did not consider the dimension of competency development through the analysis of differences in participants' levels of behavioral competencies at the beginning and at the end of the training period. Therefore, to understand if the participants' learning activities really impacted their possession of ESC competencies, we included control variables that might have influenced students' level of behavioral competencies at the beginning of our study period. Indeed, prior studies suggest that demographic characteristics, such as gender and age, may affect the possession of ESC competencies (Byron, 2007; Taylor and Hood, 2011). Since students who participated in the study were all enrolled in the first year master's degree program and thus did not show significant differences in terms of age, we included gender as a control variable. Gender was coded as 1 = male and 0 = female.

Competencies can be shaped by virtue of an individual's interaction with the environment and the experience he or she accumulates during the lifespan (Boyatzis, 1982). Thus, at the beginning of the master's course, we asked students to indicate if they had ever performed work activities. On-the-job work experiences are often seen as a primary source of individual development and learning. Through prior work, individuals not only acquire technical skills related to a specific profession, but they may also have the opportunity to learn and practice behavioral competencies that can be applied to different organizational contexts (Tesluk and Jacobs, 1998). Work experience was assessed through a dichotomous variable where “1” means that the student had prior working experience and “0,” otherwise.

## Findings

Descriptive statistics and measures of association among the variables are reported in Table 2.

**Table 2:** **Means, standard deviations, and correlations**.

	**Mean**	**Standard deviation (*SD*)**	**TL**	**IEL**	**SEL**	**Emotional**	**Social**	**Cognitive**	**Gender**
TL	0.521	0.300							
IEL	0.089	0.163	0.152						
SEL	0.157	0.178	0.119	0.488^***^					
Emotional competencies	3.894	0.383	0.112	0.050	0.049				
Social competencies	3.703	0.484	0.144	0.087	0.090	0.635^***^			
Cognitive competencies	2.909	0.866	0.083	0.090	0.073	0.404^***^	0.476^***^		
Gender	0.789	0.410	0.036	−0.034	0.034	−0.039	0.043	−0.075	
Work experience	0.726	0.448	0.004	0.193^*^	0.047	0.037	0.130	−0.001	0.204^**^

* p < 0.10;

** p < 0.05;

*** *p < 0.01*.

**Figure 1:**
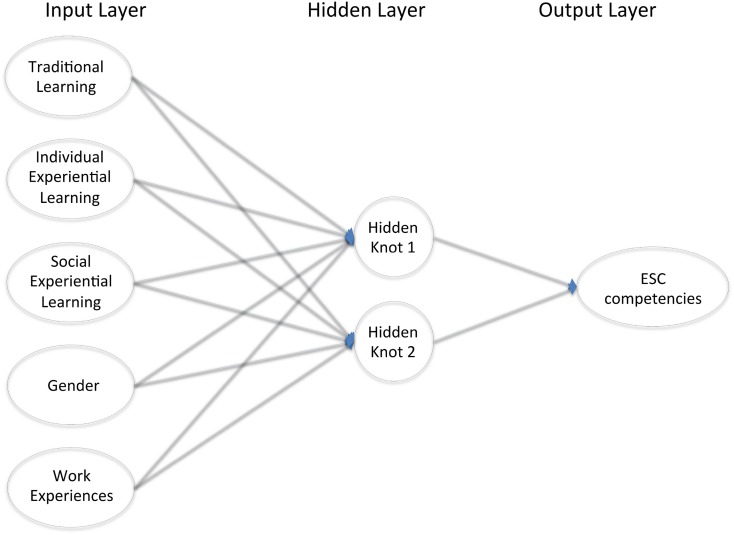
**Artificial neural network feed forward ANN(5, 2, 1)**.

To test our research hypotheses, we used the Artificial Neural Networks (Bishop, 1995; Ripley, 1996; Haykin, 1999). More precisely, since the independent variables are the three learning methods (TS, IEL, SEL), we defined three different Artificial Neural Networks (ANNs). This choice is motivated by the consideration that the ANNs are a more flexible model compared with the regression one. The ANNs, in fact, are able to capture both linear and non-linear relationships between variables without a priori definition of the type of relationship. This feature enables us also to delve into the interaction among variables, consistent with our study whose aim is to investigate the impact of the interplay between traditional and experiential learning methods on ESC competencies development. In doing so, we trained different feed-forward neural networks using 80 sample points, whereas the remaining 15 form the validation set. Concerning the structure of the ANN and due to the number of observations, we considered a neural network with one hidden layer with two nodes. To avoid the drawback of a possible arrest of the estimation algorithm at a local minimum, we repeated the estimation 100 times. Among all the estimated ANNs, we chose the one that minimized the Network Information Criterion. The difference among these ANNs is essentially on the number of nodes in the hidden layer: we considered two, three, and four hidden nodes and the choice of the best ANN was based on a penalty function computed on the validation set. Figure 1 depicts the ANN that was selected.

**Figure 2:**
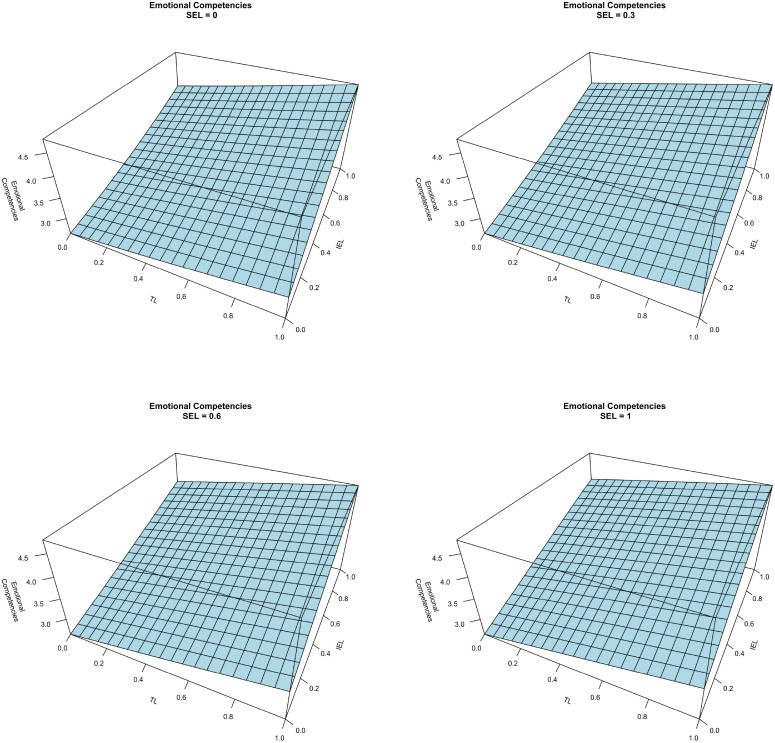
**Emotional competencies vs. TL and IEL at different level of SEL**.

**Figure 3:**
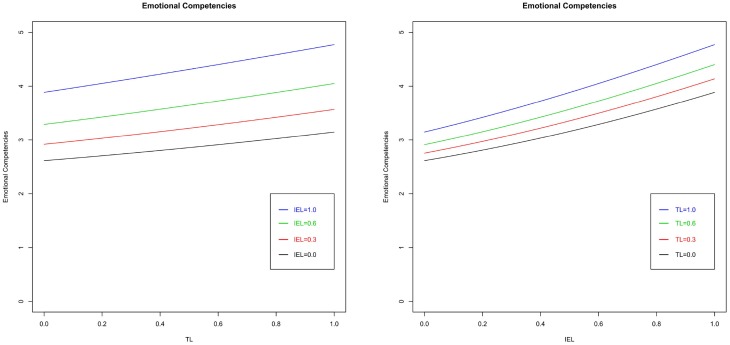
**Emotional competencies vs. TL (left) and IEL (right) at fixed level of SEL**.

**Figure 4:**
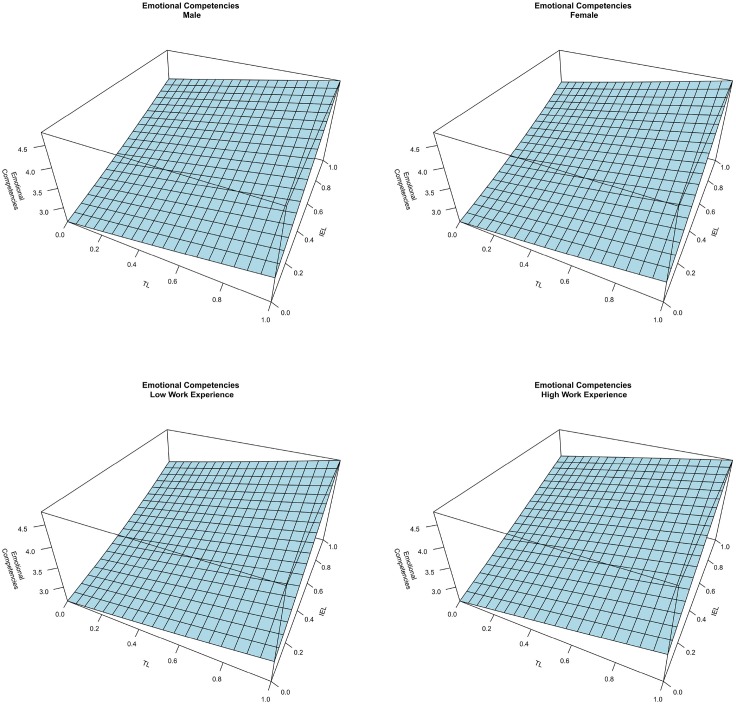
**The effect of gender and work experience on emotional competencies**.

The results of the three artificial neural networks, one for each dependent variable, are presented graphically. Considering the impact of the different learning methods on emotional competencies, Figure 2 counts four graphs, each of them considers a different level of the SEL variable (0, 0.3, 0.6, and 1). The axes of the horizontal plane are related to the other two independent variables, namely TL and IEL. The values of the independent variables are expressed as a ratio between the number of techniques a student was exposed to and the total possible number of techniques. Comparing the different graphs we saw that they do not differ from each other significantly, and this means that the SEL did not impact on emotional competencies. In addition, from the graphs a combined effect between TL and IEL on emotional competencies emerges. An increase in the numbers of both TL and IEL has a positive effect on emotional competencies. Nonetheless, these two learning modes have a different impact on emotional competencies as illustrated in Figure 3. The graphs represent the Emotional Competencies according to TL at four different levels of IEL, and according to IEL at four different levels of TL (in both cases at a fix level of SEL). Considering the graph on the left, when IEL is not adopted, the slope of the curve is higher in comparison to the maximum level of IEL (all possible techniques of this learning mode are practiced by the student). This means that the marginal incremental effect of TL on emotional competencies decreases as the IEL increases. The graph on the right represents the same combined effect of the two learning methods, but, in this case, at high levels of TL, IEL continues to increase the level of emotional competencies. Therefore, it seems that the two learning methods are required to develop emotional competencies. Thus, H1 is supported. The analysis also considers the effects of gender and working experience as control variables, which do not affect emotional competencies. Indeed, as depicted in Figure 4, the surfaces of the graphs do not change when we vary the values of the control variables.

**Figure 5:**
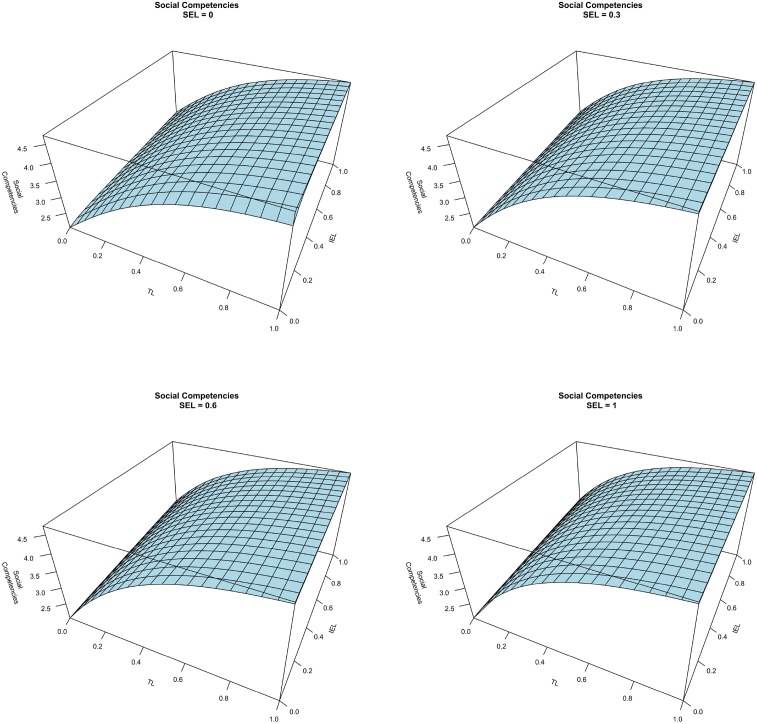
**Social competencies vs. TL and IEL at different level of SEL**.

**Figure 6:**
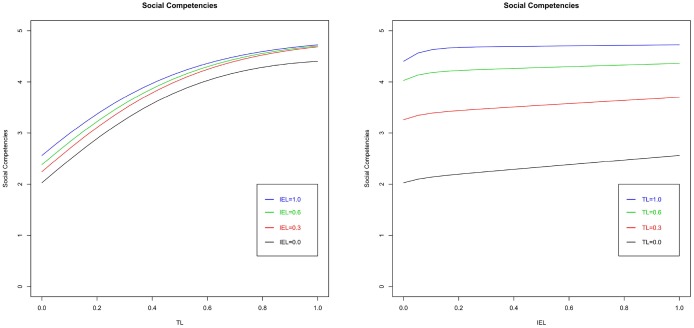
**Social competencies vs. TL (left) and IEL (right) at fixed level of SEL**.

**Figure 7:**
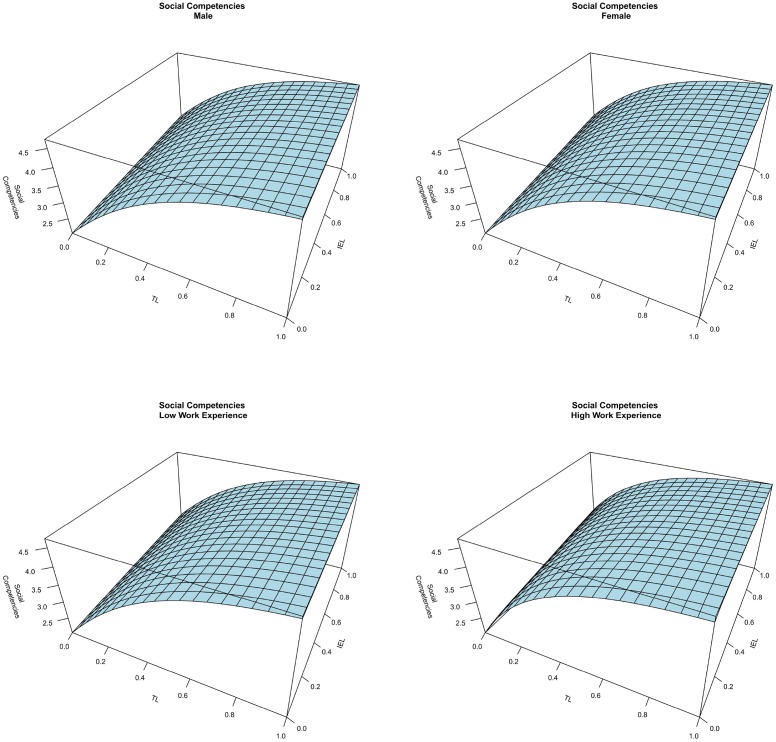
**The effect of gender and work experience on social competencies**.

Figure 5 shows four graphs of the interplay between TL and IEL on social competencies at different levels of SEL (0, 0.3, 0.6, and 1). The comparison among the four planes shows that there is a positive interplay between SEL and TL on social competencies. As seen in Figures 5, 6, the effect of TL on social competencies is not linear. Indeed, the marginal effect of TL decreases when the TL techniques approach the maximum level. Finally, findings show that IEL does not impact the level of social competencies. Thus, H2 is supported. As in the previous discussion, Figure 7 shows that the control variables do not affect social competencies.

**Figure 8:**
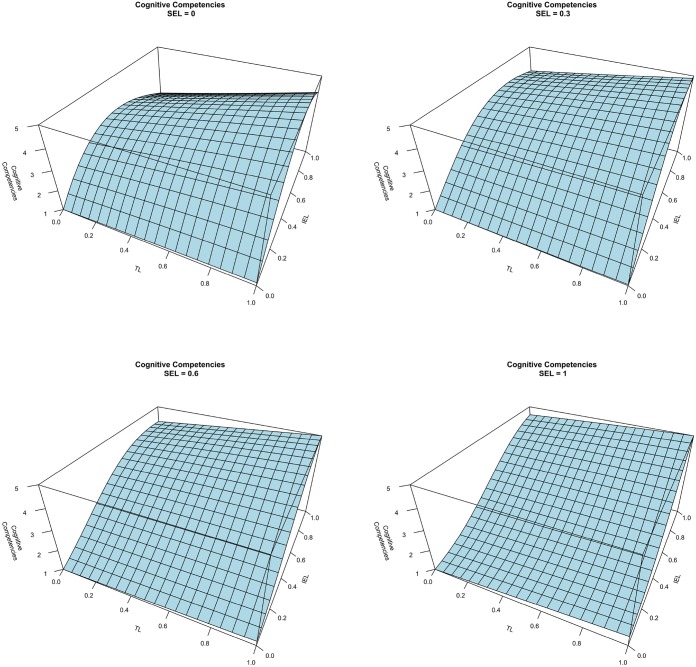
**Cognitive competencies vs. TL and IEL at different level of SEL**.

**Figure 9:**
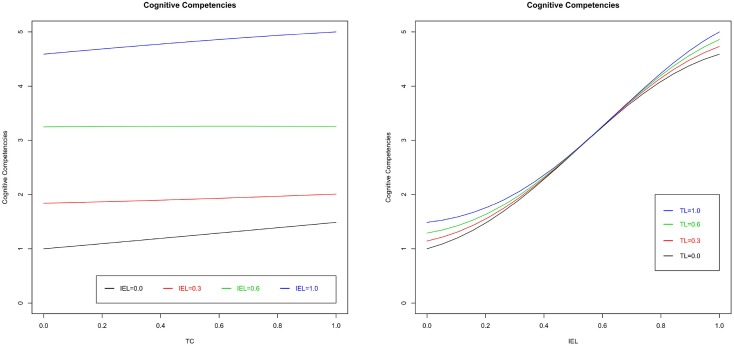
**Cognitive competencies vs. TL (left) and IEL (right) at fixed level of SEL**.

**Figure 10:**
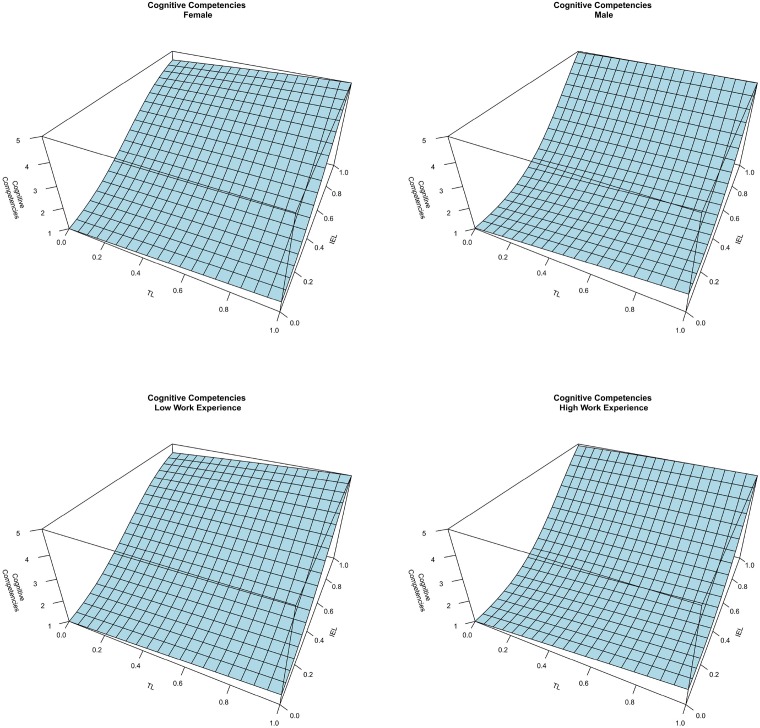
**The effect of gender and work experience on cognitive competencies**.

Finally, Figure 8 represents the combined effect between TL and IEL on cognitive competencies at different levels of SEL. TL seems to have a very limited impact on cognitive competencies, which emerges when comparing the four graphs. Figure 9 represents the Cognitive Competencies according to TL at four different levels of IEL, and according to IEL at four different levels of TL (in both cases at a fix level of SEL). These findings show that, at a fixed level of SEL, an increase of IEL positively affects the level of cognitive competencies, whereas TL has no impact on cognitive competencies. However, considering the first graph of Figure 8, when SEL is at the lowest level, findings show a positive interplay between TL and IEL. This means that while IEL is at the lowest level, TL does not impact cognitive competencies, whereas when IEL is at high levels, TL presents a positive impact on cognitive competencies. Furthermore, considering the impact of SEL, it seems that involving students in activities that require social interactions has a negative impact on systems thinking and pattern recognition, apart from high levels of IEL in which the combined effect of the two experiential learning methods shows high levels of cognitive competencies. In summary, these results highlight a prominent role of individual experimental learning method that reinforces the effect the traditional and the SEL approaches. Thus, H3 is supported only at high levels of IEL. Finally, Figure 10 illustrates that the control variables do not have any impact on cognitive competencies.

## Discussion and contribution

These findings contribute to understanding the relationship between learning methods and improvement of competencies. The paper draws attention to an issue still neglected by the extant literature, namely the interplay between traditional and experiential learning methods. Research has maintained that in-class traditional training does not represent an opportunity for competency development, which can be effectively stimulated primarily through experiential learning methods (Laker and Powell, 2011). Contrary to prior studies, our results provide counterintuitive evidence, suggesting that TL techniques need to be implemented together with IEL, on the one hand, to achieve a higher effect on emotional competencies and with SEL, on the other hand, to have an impact on social competencies. Therefore, the first result of this study suggests a combination of learning modes that can be used to stimulate the competency development process. Considering our first hypothesis, the role of TL techniques to support the understanding of emotional competencies and being aware of them, through the observation of effective examples of their manifestations, seems to be confirmed. On the other hand, social competencies can be leveraged involving individuals in experiential learning, not only through those activities that favor group interaction (SEL) but also through TL techniques, even as we found from our findings the marginal effect of using more TL techniques is decreasing. Finally, although cognitive competencies are stimulated primarily by IEL, we also found that experiential learning is not always effective for improving cognitive competencies. Indeed, the results show that the involvement of students in SEL techniques alone may be detrimental for the development of cognitive competencies, whereas TL had no impact on this type of competency. SEL and traditional methods play a role only at high levels of IEL. All these results support the importance of the combined effect of the use of these methods and their mutual reinforcement on the process of competency development.

A second result is about determining the role of these combinations of methods on specific groups of competencies. From our analysis, the techniques that involve the student individually (such as participation in seminars, talks from experts, study visits, individual project works, and practical activities) show an impact on the behavioral competencies that tend to be related to self-awareness and self-management (such as Initiative, Self-Control, Achievement Orientation, Adaptability, etc.), and even if the combination of TL and IEL methods produces great improvement, the role of IEL methods appears to be more relevant than that of TL methods. In contrast, using methods that require interaction, SEL presents no effect on the competencies related to self-awareness and self-management. On the other side, a development of competencies related to understanding and managing others (such as Empathy, Influence, Leadership, Conflict Management, etc.) may be obtained by using methods that require interactions with other participants (that is the most common way) and also by using TL methods (that is a less expected solution). This may be due to the fact that TL methods in any case imply some kind of interaction—even if limited—with a teacher or part of the class; participating in a a study visit or an interactive seminar are some examples of situations where competencies like Communication, Influence, and Organizational Awareness may be experimented with and trained. The results are even different for cognitive competencies, where IEL methods show high impact, whereby SEL and TL play a role in stimulating these types of competencies only if IEL is at its highest level. This can be ascribed to the fact that in our framework we included the only two cognitive competencies (systems thinking and pattern recognition) that prior research has demonstrated to distinguish between best and average performers, and that are specifically related to effectiveness in work and academic settings (Boyatzis, 2008, 2009). TL may have a higher impact on other cognitive competencies not investigated here that can be considered threshold, such as written communication, quantitative analysis, use of concepts, or theory building. Results also show that IEL methods combined with SEL methods produce a high increase in cognitive competencies. This means that involving students only in activities that require social interactions is not enough to stimulate the development of systems thinking and pattern recognition abilities. SEL techniques imply complex situations in which students are exposed to an amount of information provided by peers that need to be elaborated and discussed to achieve a common goal, as in cases of group project work. As suggested by prior studies (Chen et al., 2004), students may find working and learning in a group to be challenging, and, at times, quite frustrating. These cognitive competencies are performed by behaviors, such as seeing the commonality or similarities among many elements and identifying the various factors that impact upon a complex situation or event, that seem better developed through learning techniques which imply individual practice and reflection.

A third result is about the role of the control variables. From our analysis, the control variables we considered do not present any significant effect on the relationship between learning techniques and competencies development. This result is particularly important to reinforce our findings since it shows that the level of possession of ESC competencies by our sample did not depend on previous experiences or personal conditions that might have influenced them. This means that because no other stimulus had an impact on the possession of ESC competencies, these competencies have been mainly influenced by the learning methodologies experienced by the students.

Another contribution of this study concerns the classification of the learning methods proposed (TL, IEL, and SEL). Specifically, we introduced a distinction between individual and social learning within the general category of experiential methods. Our results demonstrated the importance of this distinction since the two learning approaches present different effects on the participants' competency portfolio. Finally, one additional contribution is about the adoption of the artificial neural network as a methodological approach that enabled us to unravel the combined effect of the three learning methods on each behavioral competency (emotional, social, and cognitive).

## Research implications and limitations

These findings also offer some implications in terms of higher education and management development. At the institutional level the necessary changes of the academic educational programs are invoked, as demonstrated, for instance, by the initiatives that aim to implement a whole-person or student-centered learning approach within the higher educational programs (Hoover et al., 2010). These programs can be designed in such a way that learners can have the opportunity to assess and become aware of the behavioral competencies they possess and of those that are considered useful and necessary for academic disciplines as well as for professional and vocational areas (Villa Sánchez and Poblete Ruiz, 2008). Competency-based learning objectives should not only be integrated in the academic curricula through the design of dedicated courses, but they can also be included into existing courses devoted to technical skills acquisition. Therefore, our results provide insights on how competency development can be complemented in technical training rather than supplanting it.

In order to maximize competency development in existing courses, learning activities need to be organized differently. First, the results of the combined effect of the three learning modes on emotional, social, and cognitive competencies can be considered by instructors while organizing the curriculum and restructuring their courses around integrative behavioral skills and defining the most effective pedagogical tools to be adopted. In this regard, instructors need to be supported as they increase their awareness on how their courses (contents and didactic techniques) may have an impact not only on the acquisition of professional knowledge and skills but also on the development of behavioral competencies.

Second, instructor's awareness concerning the importance of ESC competencies and how to stimulate their acquisition within existing course needs to be translated into explicit learning objectives to avoid the “hidden curriculum” in which the concepts of ESC competencies are not never fully explained in class, but are implicitly transferred through the application of a learning technique such as group exercises (Bedwell et al., 2014). It is important that learners understand what is expected of them through the definition of specific learning objectives that target technical as well as emotional, social and cognitive skills. Not only do explicit learning outcomes allow participants to set expectations but they also allow instructors to select the appropriate learning methods and to design coherent and effective assessment tools (Bedwell et al., 2014).

Finally, since behavioral competencies are more complex and difficult to teach than disciplinary subjects, pursuing their development in educational programs can face obstacles. Implementing different learning approaches is time consuming and personally demanding for instructors that need to combine their traditional roles as experts with the new role of learning facilitators that gives more space for participants' reflection and interaction. Therefore, instructors may require different attitudes and mindsets to confidently blend traditional and experiential learning approaches into their courses.

Beyond suggesting these substantive changes in the academic curriculum, our research also offers implications in terms of competency development at the organizational level. When firms invest in training activities with the aim to develop the competency profile of future leaders, they should select the educational programs by considering the impact of the different learning methods adopted. Companies may analyze their specific needs, defining the emotional, social, or cognitive competencies that need to be improved in their employees. Consequently, the training programs and the related teaching methods will be selected accordingly.

This study also presents some limitations, which may constitute opportunities for further research. First of all, future studies can include the analysis of pre and post-test differences in the research design to evaluate the level of behavioral competencies at the beginning and at the end of the training period. Another limitation is that this study is not longitudinal and, for this reason, does not consider the dimension of competency development that occurred during the entire duration of the master's degree programs, focusing only on the relationship between the learning activities in which participants were involved during a semester and the level of possession of behavioral competencies at the end of this period. However, in our study we considered some variables—gender and prior experience—as possible factors that might have influenced the level of ESC competencies of the participants and we did not find any significant effect of these controls on the level of possession of behavioral competencies. The results of this analysis support the relevance of learning methods and their interplay as they influence competency development. In addition, the courses included in our study are part of the curriculum of master's degree programs that are all innovative in some respects, both in terms of contents and didactic methods used. During their 2-year program, the participants attend courses that have adopted similar learning approaches. A further promising line of research should consider differences in attained competency by investigating innovative courses that already blend traditional and experiential learning approaches and courses that continue to rely on traditional in-class lectures as the main teaching method. This study could also be complemented by a performance analysis conducted after participants' entry into the labor market. In this regard, future research should hypothesize that the learning methods and the subsequent competency would pay off in terms of better job placement and career progression. Furthermore, research should take into account the factors related to the learning climate, like instructor's style, classroom composition and size, which may influence the participants' motivation and effort in the didactic activities proposed as well as the extra didactic experiences that the academic institution can provide to the students. Other individual variables could be of interest as potential predictors of differential improvement in ESC competencies; for instance, some personality types may respond more favorably to the experiential learning approach than others.

In conclusion, this research has contributed to the ongoing debate on the role of higher education to equip graduates and executives with the competencies required by the labor market to increase the level of productivity and innovation in the workplace. It would be valuable to explore how ESC competencies development can be integrated with existing courses and how the combined effect of traditional and experiential methods can maximize learning outcomes.

### Conflict of interest statement

The authors declare that the research was conducted in the absence of any commercial or financial relationships that could be construed as a potential conflict of interest.
