# Supplementary material for: The interplay between experiential and traditional learning for competency development
Source: Front Psychol. 2015 Sep 2;6:1305. doi: 10.3389/fpsyg.2015.01305 (PMC4557282; doi:10.3389/fpsyg.2015.01305)
Supplement: Supplementary file 1 [file Appendix.PDF]

APPENDIX

The Emotional Competence Inventory-University Version (Boyatzis and Goleman, 2002)

Self-Awareness concerns knowing one's internal states, preferences, resources, and intuitions. The Self-Awareness cluster contains three competencies:

- Emotional Awareness: Recognizing one's emotions and their effects
- Accurate Self-Assessment: Knowing one's strengths and limits
- Self-Confidence: A strong sense of one's self-worth and capabilities

Self-Management refers to managing ones' internal states, impulses, and resources. The Self-Management cluster contains seven competencies:

- Emotional Self-Control: Keeping disruptive emotions and impulses in check
- Trustworthiness: Maintaining integrity, acting congruently with one's values
- Conscientiousness: Taking responsibility for your personal performance
- Adaptability: Flexibility in handling change
- Achievement Orientation: Striving to improve or meeting a standard of excellence
- Initiative: Readiness to act on opportunities
- Optimism: Persistence in pursuing goals despite obstacles and setbacks

Social Awareness refers to how people handle relationships and awareness of others' feelings, needs, and concerns. The Social Awareness cluster contains three competencies:

- Empathy: Sensing others' feelings and perspectives, and taking an active interest in their concerns
- Organizational Awareness: Reading a group's emotional currents and power relationships
- Service Orientation: Anticipating, recognizing, and meeting customers' needs

We included a fourth competency, Cultural Awareness, presented in the previous version of ECI, namely appreciating and understanding people with different backgrounds and culture.

Relationship Management concerns the skill or adeptness at inducing desirable responses in others. The Relationship Management cluster contains eight competencies:

- Developing Others: Sensing others' development needs and bolstering their abilities
- Inspirational Leadership: Inspiring and guiding individuals and groups
- Change Catalyst: Initiating or managing change
- Influence: Wielding effective tactics for persuasion
- Conflict Management: Negotiating and resolving disagreements
- Teamwork: Working with others toward shared goals. Creating group synergy in pursuing collective goals
- Communication: Sending clear and convincing messages to an audience in an open and effective way
- Building Bonds: Developing and maintaining good relationships with a variety of people

Cognitive competencies: intellectual abilities that are related to people's effectiveness.
The Cognitive competencies cluster contains two competencies:

- Systems Thinking: Identifying the many and various factors that impact upon a complex situation or event
- Pattern Recognition: Recognizing patterns or trends in random information, events or situations
